# Expanding the Armamentarium of Donor Sites in Microvascular Head and Neck Reconstruction

**DOI:** 10.3390/jcm13051311

**Published:** 2024-02-26

**Authors:** Z-Hye Lee, Ana Canzi, Jessie Yu, Edward I. Chang

**Affiliations:** Department of Plastic Surgery, University of Texas MD Anderson Cancer Center, 1400 Pressler Street, Houston, TX 77030, USA

**Keywords:** head and neck reconstruction, microvascular reconstruction, workhorse-free flaps

## Abstract

The field of microsurgical head and neck reconstruction has witnessed tremendous advancements in recent years. While the historic goals of reconstruction were simply to maximize flap survival, optimizing both aesthetic and functional outcomes has now become the priority. With an increased understanding of perforator anatomy, improved technology in instruments and microscopes, and high flap success rates, the reconstructive microsurgeon can push the envelope in harvesting and designing the ideal flap to aid patients following tumor extirpation. Furthermore, with improvements in cancer treatment leading to improved patient survival and prognosis, it becomes increasingly important to have a broader repertoire of donor sites. The present review aims to provide a review of newly emerging soft tissue flap options in head and neck reconstruction. While certainly a number of bony flap options also exist, the present review will focus on soft tissue flaps that can be harvested reliably from a variety of alternate donor sites. From the upper extremity, the ulnar forearm as well as the lateral arm, and from the lower extremity, the profunda artery perforator, medial sural artery perforator, and superficial circumflex iliac perforator flaps will be discussed, and we will provide details to aid reconstructive microsurgeons in incorporating these alternative flaps into their armamentarium.

## 1. Introduction

Microvascular head and neck reconstruction has advanced tremendously over the years with high flap success rates often over 95% at most high-volume centers [1,2,3]. With the high success rates that can be achieved in the current era, the reconstructive demands have also increased. The goals of reconstruction have progressed well beyond simply preventing thrombosis and total flap loss. The microsurgeon is now more than ever tasked with optimizing the aesthetic and functional outcomes for patients undergoing tumor extirpation. While the overwhelming majority of defects can be reliably reconstructed using the standard workhorse flaps such as the radial forearm, the anterolateral thigh (ALT), and the latissimus dorsi flap, circumstances can arise where alternate donor sites may be necessary. With the improvements in cancer treatment and patient survival, patients may develop recurrent disease, develop complications following radiation, or may undergo such extensive resections that multiple flaps are needed [4]. As such, the reconstructive microsurgeon should become familiar with alternate donor sites in the setting that the standard workhorse donor sites are unavailable [5].

While the radial forearm remains one of the most reliable flaps with a long pedicle and provides thin pliable tissue, the need for a skin graft to the donor site or radial dominant perfusion to the hand may necessitate an alternative donor site. In the Western population where an increasing body mass index and obesity are increasingly common, the ALT may be prohibitively thick, while in other circumstances, when patients are malnourished and suffer significant weight loss, perhaps donor sites with more volume are warranted. The present article aims to provide a review and synopsis of some donor sites that have been gaining popularity and may benefit surgeons performing high-volume microvascular head and neck reconstruction. The review aims to provide a synopsis of the ulnar forearm, lateral arm, profunda artery perforator (PAP), medial sural artery perforator (MSAP), and superficial circumflex iliac perforator (SCIP) flaps, focusing on anatomy and pearls and pitfalls to the utilization of these flaps in head and neck reconstruction.

## 2. Ulnar Forearm Flap

While the radial forearm continues to remain one of the most popular donor sites, in circumstances when the patient is radial dominant or perhaps arterial catheters have been placed into the radial artery, a flap based on the ulnar artery is a reliable option to consider. However, in other circumstances, one may opt to use the ulnar donor site as the primary option to design a flap with slightly more volume or harvest a flap that is less hair-bearing. In these circumstances, an Allen test should also be performed to ensure that perfusion to the hand will not be compromised with the sacrifice of the ulnar artery [6].

The ulnar forearm flap can be harvested distally just proximal to the wrist crease, similar to the design of a radial forearm flap. The artery is readily palpable or can be identified with a handheld Doppler to orient the flap so that it is centered over the ulnar artery. The flap is raised as a fasciocutaneous flap, again similar to the harvest of the radial forearm flap. A distally based ulnar fasciocutaneous flap will provide a longer pedicle; however, designing the flap more distally in this fashion will lead to exposure of the flexor digitorum superficialis (FDS) and the flexor carpi ulnaris (FCU) tendons that can become exposed in the setting that the skin graft has poor take [7]. The flap harvest should therefore aim to preserve the fascia over the tendons to minimize wound healing complications of the skin graft over the tendons.

**Figure 1:**
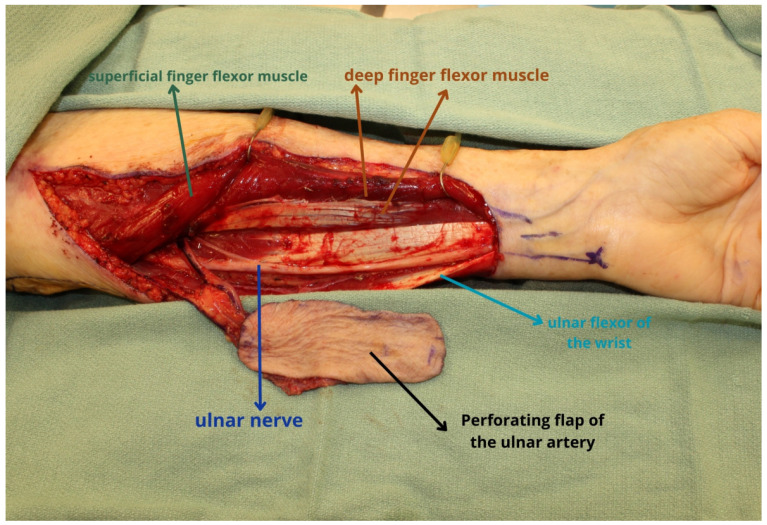
The ulnar artery perforator flap is based on perforators arising from the ulnar artery and is harvested more proximally to avoid issues with tendon exposure in the setting of poor skin graft take. As noted, the flap is on the ulnar aspect of the forearm which is often less hair-baring, but the pedicle length is shorter than the radial forearm flap.

**Figure 2:**
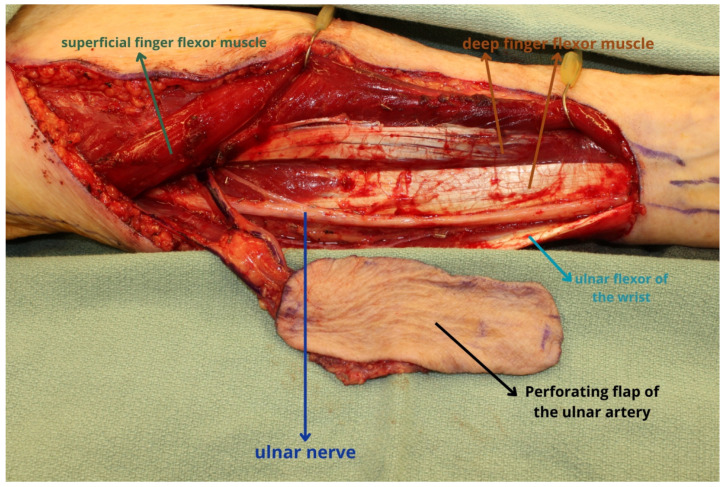
During dissection, it is critical to avoid injury to the ulnar nerve which runs in close proximity to the pedicle as depicted. The soft tissue flap is ideally suited for the reconstruction of partial glossectomy, buccal mucosal, or palatal defects. The present flap was utilized for reconstruction of a partial glossectomy defect. Dissection of the pedicle can be performed more proximally to gain additional pedicle length, but flap selection should consider the availability of recipient vessels.

Alternatively, the flap can be harvested as a perforator flap which has also been proven to be extremely reliable. The ulnar artery perforator (UAP) flap is harvested more proximally than its fasciocutaneous counterpart since the perforators arise more proximally [8,9]. The distal extent of the flap is typically oriented approximately five centimeters proximal to the pisiform along an axis from the pisiform to the medial epicondyle (Figure 1). By harvesting the flap more proximally, the average pedicle length is approximately 7.1 cm; however, this reduces the risks of tendon exposure and compromised take of the skin graft. Perforators have been reliably mapped using the pisiform as a landmark where the A, B, and C perforators arise 7 cm, 11 cm, and 15 cm from the pisiform, respectively. The flap dissection starts on the radial side and is harvested in a suprafascial plane until the dissection proceeds to the FDS and FCU tendons from the radial and ulnar sides, respectively. At this time, the dissection must proceed in a subfascial plane to include the pedicle and perforators in the flap. During the dissection, careful attention must be paid to avoid injury to the ulnar nerve which is intimately adjacent to the ulnar vessels (Figure 2).

Studies examining outcomes using an ulnar forearm flap have proven to have equally high success rates and equivalent post-operative speech and swallowing function when compared to the radial forearm flap [10]. Not surprisingly, studies examining donor site morbidity have also demonstrated similar risks of complications compared to the radial forearm donor site [11,12,13]. Some studies have even found superior outcomes with the ulnar forearm donor site compared to its radial counterpart [14].

## 3. Lateral Arm and Lateral Forearm Flap

**Figure 3:**
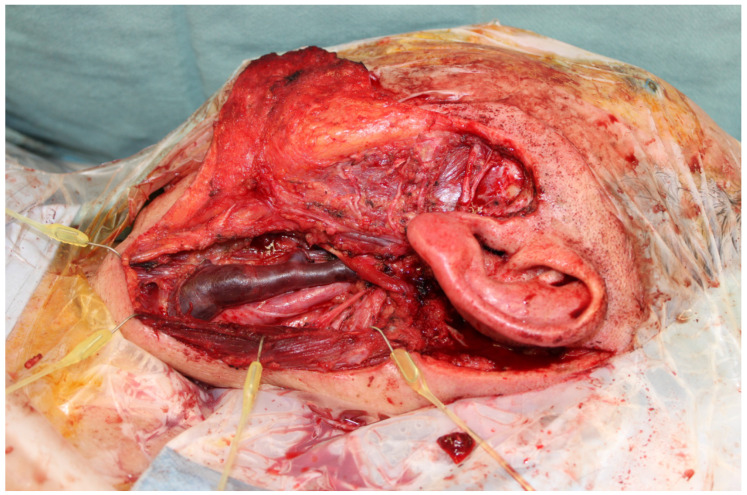
Parotidectomy defect with preservation of the facial nerve but with a significant skin resection. The lateral arm donor site often provides a suitable color match to the facial skin and can be harvested more proximally or distally based on the thickness needed.

**Figure 4:**
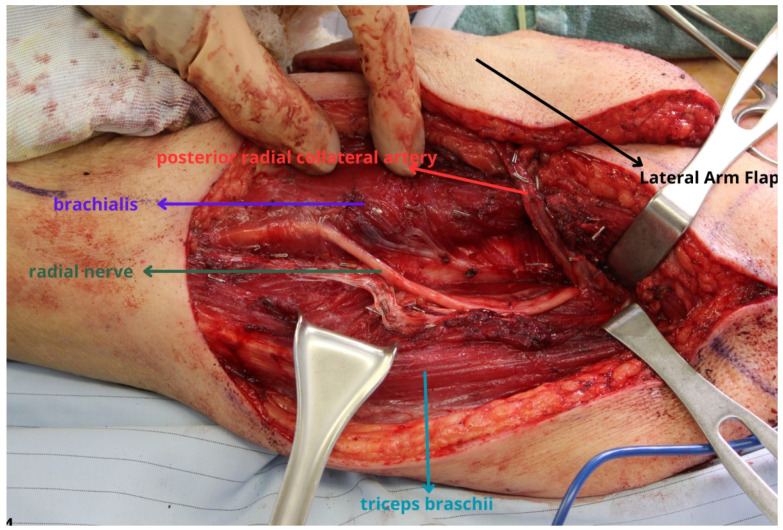
The radial nerve lies in close proximity to the pedicle and must be carefully protected during dissection. The patient’s thigh was too thick to use for the parotidectomy defect which is more common in the Western population. The lateral arm flap is well-suited as a flap that is often intermediate in thickness. Designing the flap more proximally as shown will provide more thickness while a more distally oriented flap will provide thinner tissue but allow for a longer pedicle. The design of the flap can be adjusted based on the extent of the defect and the need for more volume.

The lateral arm represents a unique donor site option that permits the harvest of a flap of variable thickness that can be tailored based on the extent of the defect [15]. For many head and neck defects, the lateral arm also provides a comparable color match to facial skin compared to the thighs (Figure 3). Similar to the ulnar forearm donor site, a lateral arm flap can be harvested as a fasciocutaneous flap or a perforator flap. Also like the ulnar forearm flap, a true lateral arm perforator flap has a shorter pedicle compared to a more distally oriented fasciocutaneous flap. While the flap can be harvested more proximally as a perforator flap or more distally as a fasciocutaneous flap, the microsurgeon should be cognizant of the relatively smaller caliber artery which is typically less than 2 mm as well as the proximity of the radial nerve to the pedicle (Figure 4).

The lateral arm perforator (LAP) flap is harvested from the upper lateral arm and is a true perforator flap. The perforator anatomy is remarkably reliable, similar to other perforator flaps that have been described [16]. Using the landmarks of the deltoid insertion and the lateral epicondyle, the perforator locations can typically be found 7 cm, 10 cm, and 12 cm from the deltoid insertion. The flap dissection should begin from the posterior side. The dissection should be subfascial progressing from posterior to anterior towards the septum between the triceps and biceps muscles. The flap is often of an intermediate thickness between the ALT and a forearm-based flap [17]. Another advantage of the LAP flap is the opportunity to create a sensate flap as the lateral antebrachial cutaneous nerve often needs to be divided during perforator and pedicle dissection. As a trade-off, there can be an area of numbness in the donor site along the dermatomal distribution of the nerve. The LAP pedicle is considerably shorter compared to a more distally based fasciocutaneous flap which will be discussed later. On average, the length of the pedicle is approximately 7 cm, and the reconstructive microsurgeon should be conscious of the pedicle length during flap selection [16].

A more distally based lateral arm flap has been named the extended lateral arm flap or the lateral forearm flap which is a more distally based flap that can be taken over the lateral epicondyle [18]. A flap harvested this distally is often very thin and pliable and may be as thin or thinner than a traditional forearm-based flap [19]. The more distally oriented fasciocutaneous flap is centered over the distal extent of the vascular pedicle which significantly increases the pedicle length. The maximum width of the flap that can be harvested is based on the “pinch test” but is typically less than 6 cm to allow for primary closure of the donor site. Consequently, an added benefit of the lateral forearm is that it allows for the harvesting of a thin, pliable flap without the need for a skin graft to the donor site. Similar to the LAP, the cutaneous nerve can also be harvested with the flap to create a sensate flap, or the nerve can also be preserved in many circumstances to avoid numbness in the lateral arm dermatome.

The closure of the donor site for the lateral arm flaps should be carried out without tension and without re-approximation of the muscle or closure of the deep layers. A tight closure can result in radial nerve palsy with post-operative swelling that has catastrophic consequences if the radial nerve deficits do not resolve [20]. Thus, even if the flap harvest is performed using a no-touch technique, paying careful attention to protect the nerve, a tight closure can still result in neurologic deficits. While suboptimal, a skin graft to the donor site is preferable to a radial nerve deficit.

## 4. Profunda Artery Perforator Flap

**Figure 5:**
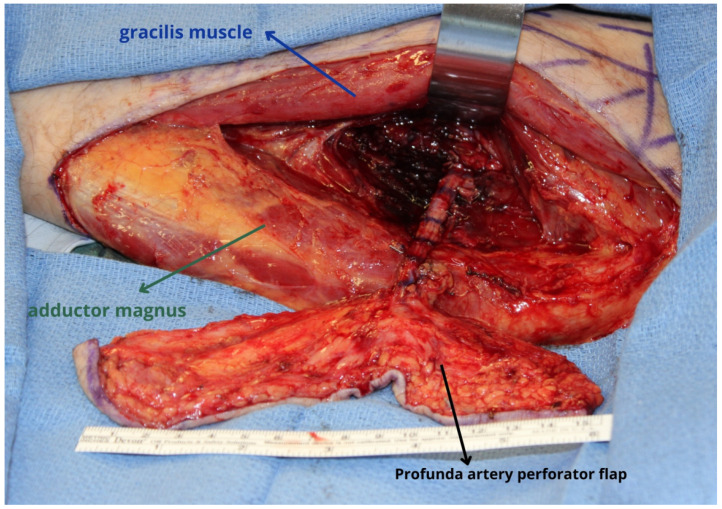
Profunda artery perforator flap harvested based on perforators arising from the profunda femoris artery with suitable pedicle length when the pedicle is dissected to its origin from the profunda femoris artery. To gain a longer pedicle and a larger caliber artery, the dissection should be performed to the takeoff from the profunda femoris artery. Retraction of the gracilis muscle is necessary to perform the more proximal dissection to the origin of the pedicle.

**Figure 6:**
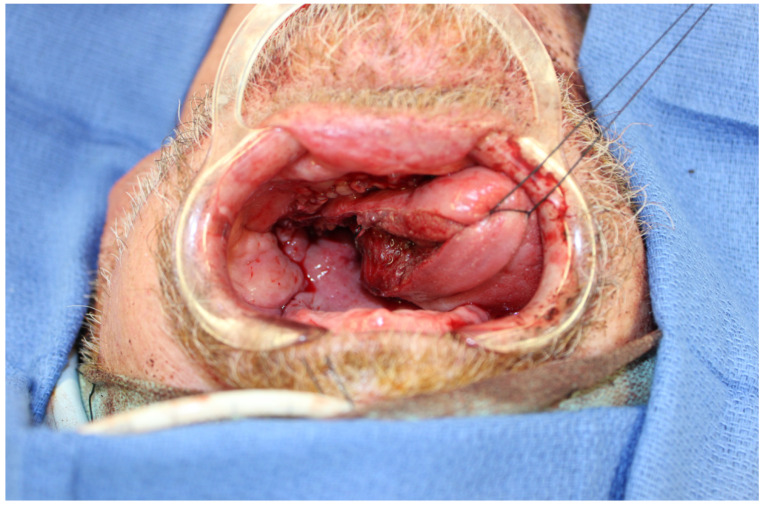
A defect that was reconstructed using the MSAP flap. Hemiglossectomy defect that could be reconstructed with any number of potential donor sites. However, using an upper extremity flap often requires sequential harvest after the resection is completed. Using a thinner donor site from the lower extremity allows for simultaneous harvest and can shorten the operating time.

The profunda artery perforator (PAP) flap has been popularized in the United States as the secondary workhorse flap for autologous breast reconstruction [21,22], but the donor site also represents a reliable option for the reconstruction of head and neck defects [23]. While most PAP flaps performed for breast reconstruction are typically harvested in a transverse orientation, the perforator anatomy is much more reliable in a vertically oriented flap. The perforator anatomy again has proven to be remarkably reliable and likely more reliable than the ALT, which can have tremendous variation and in some circumstances may not have any suitable perforators at all. For many head and neck patients who suffer from weight loss due to the extensive tumor burden, pain, trismus, or the sequelae of previous radiation, the PAP can provide tissue that is thicker than the ALT (Figure 5 and Figure 6) [24,25,26]. The length of the flap can be tailored to the size of the defect, but the width that can be harvested is variable from patient to patient and is dependent on the laxity in the donor site. In some instances, flaps as wide or wider than 10 cm can be harvested while still allowing for primary closure of the donor site.

Based on the groin crease, perforators again can be reliably found at 8 cm, 13 cm, and 18 cm from the crease [27]. While the proximal-most perforator is often present, it is often not the largest perforator which tends to be more distal. Therefore, if one plans to harvest the flap in a transverse orientation, imaging studies are recommended for the novice microsurgeon to confirm the presence of a suitable perforator to allow the harvest of a transverse PAP flap. The dissection begins from the anterior edge of the flap and should proceed posterior to the gracilis muscle where the fascia overlying the adductor magnus is incised to identify the perforators. Since the perforators tend to arise from the profunda femoris vessels in a segmental fashion, the microsurgeon should be aware that it is difficult to design a PAP with two separate skin islands. Harvesting a chimeric PAP is possible by taking a portion of the adductor magnus if a large muscle branch is identified arising from the same perforator. In contrast to other chimeric flaps, the fasciocutaneous skin component and the muscle are typically very close to each other, which limits and restricts the mobility of each component.

The pedicle length tends to be somewhat deceptive as the dissection is performed through an intramuscular course to its takeoff from the source vessels. The trajectory can result in a pedicle that nears 12–15 cm in situ; however, upon ligation of the vessels, the pedicle length tends to retract considerably and often only provides a pedicle length of up to 8–10 cm. The artery is generally smaller compared to the ALT, typically approximately 2 mm, while the vein is usually a reasonable caliber similar to the ALT.

## 5. Medial Sural Artery Perforator Flap

**Figure 7:**
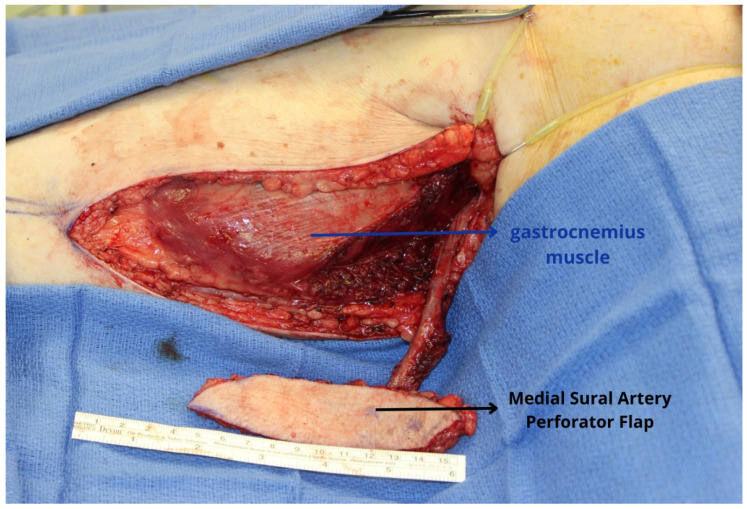
The medial sural artery perforator flap harvested from the calf region provides a suitably thin, pliable tissue to reconstruct the hemiglossectomy defect. The pedicle length is quite variable with the MSAP flap, and while a 10–12 cm pedicle is possible, this is rather inconsistent from one patient to another.

The medial sural artery perforator (MSAP) flap is another alternate donor site that is gaining popularity [28,29]. While it is more commonly used for limb salvage and extremity reconstruction, the flap also represents a potential donor site when thinner tissue is needed for head and neck reconstruction (Figure 6 and Figure 7). While the tissue may be somewhat thicker than an upper extremity flap, the tissue is still typically thinner and more pliable than the thigh [30,31,32]. One potential advantage of the MSAP over the upper extremity flaps is the opportunity for simultaneous harvest in conjunction with the resection [32]. Thus, a two-team approach can reduce the operative time whereas harvest of a forearm flap or the lateral arm is generally difficult to perform at the same as with the resecting team. Unfortunately, flap harvest is limited to a width of approximately 6 cm but can vary based on the patient’s body habitus to allow for primary closure of the donor site. While a skin graft may be suboptimal, a tight closure should be avoided to avoid the risks of compartment syndrome.

The greatest limitation of the MSAP is the freestyle nature of the flap harvest [33]. While recommendations use the landmarks of the midline of the popliteal crease and the medial malleolus 8–18 cm along this axis is marginal [34,35], most microsurgeons still advocate using a hand-held Doppler to locate perforators before making the skin incision; however, an ultrasound can greatly simplify the flap design and harvest. Most times, the perforators are located posterior to the axis, but due to the freestyle nature of this flap, there is a possibility that the perforators may be located anteriorly. Some have performed endoscopic-assisted MSAP harvests to visualize the perforator using a minimally invasive incision to aid in flap design [36].

The location of the perforator will dictate the pedicle length, which, again, can be quite variable, but for a distally located perforator, pedicle lengths of 10–12 cm can be harvested. Another reflection of the freestyle nature of the flap is the vessel caliber, which, again, is quite variable. If the perforator arises from the main medial sural artery, the vessels are sizable, but often the perforator may arise from a secondary branch of the main medial sural vessels, leading to vessels that are on average less than 2 mm in diameter. In a virgin neck, an ample number of potential recipients are available, and selection of the superior thyroid artery or another smaller caliber artery may represent a more suitable recipient than one that creates an unfavorable size mismatch [37].

## 6. Superficial Circumflex Iliac Perforator Flap

Finally, the superficial circumflex iliac perforator (SCIP) flap is another donor site that is gaining popularity for head and neck reconstruction [38]. The SCIP or groin flap has long been a workhorse flap for reconstructive surgeons and has been popularized for extremity reconstructive and limb salvage [38,39,40]. With the increased comfort and understanding of the anatomy, indications for the SCIP are rapidly expanding. Its use is well-documented in extremity reconstruction, particularly diabetic foot wounds as well as in the growing field of lymphedema surgery; however, its utility in head and neck reconstruction remains to be elucidated. The majority of cases have used the SCIP for the reconstruction of relatively smaller intraoral defects such as for partial glossectomy or buccal mucosal defects [41,42,43,44].

**Figure 8:**
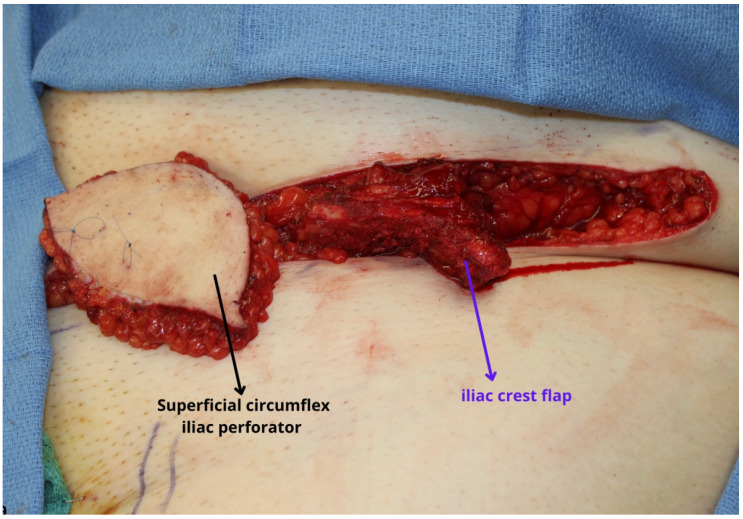
The skin paddle overlying an iliac crest flap is challenging to include with the bone, but in the setting that a skin paddle cannot be harvested with the bone based on the deep circumflex iliac artery (DCIA), a fasciocutaneous flap can be harvested based on the superficial circumflex iliac artery demonstrated here. The thickness of the superficial circumflex iliac perforator (SCIP) flap is variable based on the patient’s body habitus and may be thicker in the Western population. While difficult to appreciate in the figure, the pedicle length and caliber of the vessels are much smaller compared to other flaps and should be considered by the reconstructive surgeon prior to using this flap.

In the hands of experienced microsurgeons, the SCIP flap provides reliable thin tissue that can also be tailored as a super-thin flap or a thicker flap if both the deep and superficial branches of the superficial circumflex iliac vessels are incorporated. With the increased comfort in flap design and dissection, some authors have expanded the utility of the flap by including a portion of the iliac crest, thereby creating another option for an osteocutaneous flap (Figure 8) [45,46]. The flap can be harvested in conjunction with the resection, thereby shortening the operative time, and has minimal donor site morbidity as the donor site can be closed primarily without the need for a skin graft, leaving a well-concealed scar in the inguinal region. However, while the use of the SCIP is gradually expanding, the anatomy and dissection can pose some challenges to the novice microsurgeon. For many, the incorporation of preoperative or intraoperative ultrasound to define the vascular anatomy has greatly simplified flap harvest [47,48]. Other limitations of the SCIP flap include factors such as the pedicle length, which is relatively shorter with an average length of approximately 5 cm, and the caliber of the vessels, particularly the artery, which tend to be considerably smaller compared to some other donor sites, occasionally only one millimeter in size.

## 7. Discussion

The field of reconstructive microsurgery has revolutionized the treatment of cancer where patients previously destined for palliation, amputation, or lifelong disfigurement can now be reconstructed with high success rates and minimal complications. With the advances in technology and increased experience with microsurgery, the reconstructive microsurgeon now must consider the functional and aesthetic outcomes rather than simply focusing on flap survival. Furthermore, with the tremendous gains in cancer care, patients previously not considered surgical candidates are now able to undergo tumor extirpation with curative intent, and the onus falls on the reconstructive surgeon to optimize their quality of life. Along the same vein, modern oncologic treatments have also improved prognosis and increased patient survival, so the microsurgeon can be expected to encounter more patients who either develop recurrence or need to undergo salvage operation with another free tissue transfer. Similarly, with improved survival, patients may also suffer late complications such as hardware exposure and again need another microvascular reconstruction [49,50,51]. Under these circumstances, the reconstructive surgeon must have a broader armamentarium of flap options to be able to reconstruct these secondary defects and provide the most optimal functional and aesthetic outcomes for patients.

While there is little debate that the radial forearm, ALT, and latissimus dorsi flaps are the traditional workhorse flaps, there are also limitations to consider. The need for skin grafting and donor site morbidity of the radial forearm and the unpredictable perforator anatomy of the ALT are common complaints at both donor sites, while the latissimus dorsi typically requires harvest in a lateral decubitus position. In contrast, the alternative options presented can all be closed primarily, obviating the need for a skin graft except for the ulnar forearm donor site, although, in certain circumstances, even the ulnar forearm can be closed primarily. The PAP perforator anatomy has proven to be more reliable and consistent compared to the ALT. However, while there are certainly advantages and benefits to these alternative flaps, there are also significant limitations that must be considered.

For the majority of the alternate donor sites, the pedicle length is usually shorter, and the caliber of the artery is typically smaller when compared to the radial forearm, ALT, or latissimus dorsi flaps [5]. The vein however is typically an adequate size comparable to the diameter of the main workhorse flaps. Unfortunately, the size of the vessels cannot be modified, but the length can potentially be adjusted by harvesting and designing a more distal flap as in the ulnar forearm or the lateral arm to obtain a longer pedicle. In the setting of a redo sequential free flap or in the setting of salvage after total flap loss, a longer pedicle to reach more distant recipient vessels may be necessary to avoid the need for a vein graft. In certain circumstances, a vein graft cannot be avoided, but efforts should be made to avoid them if possible, given the higher risks of complications [52,53].

Perhaps the greatest area of consideration is whether these alternate flaps should replace the current workhorse flaps. With the increased experience and comfort with perforator flaps and smaller caliber vessels, the success rates of alternate flap donor sites and workhorse flaps are equivalent. Given the equivalent success rates, consideration perhaps should be given to using the alternate flaps as a first-line option, thereby preserving the traditional workhorse flaps in the setting of recurrent disease, flap loss, or post-operative complications. In the virgin neck when a plethora of recipient vessels are available, the shorter pedicle length that is often the Achilles’ heel of the alternate flaps becomes less of an issue. Perhaps in the current era of microvascular head and neck reconstruction, a paradigm shift may be warranted to consider using the lateral arm, PAP, MSAP, or SCIP flaps as the primary means of reconstruction. At the authors’ institution, this is becoming an increasingly popular approach where the lateral forearm flap has largely supplanted the radial or ulnar forearm flaps to avoid donor site morbidity and the need for skin grafting.

Ultimately, the reconstructive surgeon must consider all factors when discussing the available donor site options with patients. The reconstructive surgeon must assess the available donor sites, which can vary tremendously in different patient populations as Western populations tend to be more obese, precluding them from certain donor sites that are more common in Asia. Aside from the donor site itself, the surgeon must also consider the defect and select the most appropriate flap to achieve the best possible outcome. For instance, for an extensive through-and-through defect, if a flap with multiple components is needed, the ALT still represents the most reliable option. The ALT can be harvested, potentially with multiple skin paddles, and can easily also include muscle if necessary [54]. While a chimeric flap can also be designed for the PAP or the MSAP, this is often more challenging and limited due to the restrictions in mobility and length that can be obtained for each chimeric component. Finally, the surgeon must have the insight and experience to determine which donor site can be used safely and reliably. For a surgeon who has never performed one of the alternative flaps and has a complex defect in a previously operated and radiated neck, perhaps using a traditional workhorse flap would be the most prudent.

## 8. Conclusions

Microvascular head and neck reconstruction is an exceedingly challenging subspecialty in microsurgery that forces the surgeon to incorporate all of the principles of reconstructive surgery to optimize the patient’s aesthetics and function. While most defects can be successfully reconstructed with a limited number of free flap donor sites, alternative flaps can expand the armamentarium of reconstructive options in the setting of salvage surgery, flap loss, or when the traditional workhorse flaps are not available.

## Data Availability

No new data were created in the production of this work.
